# The influenza virus NS1A binding protein gene modulates macrophages response to cytokines and phagocytic potential in inflammation

**DOI:** 10.1038/s41598-020-72342-7

**Published:** 2020-09-17

**Authors:** Georgina Hotter, Chrysoula Mastora, Michaela Jung, Bernhard Brüne, Teresa Carbonell, Claudia Josa, Juan Ignacio Pérez-Calvo, Josep Maria Cruzado, Roser Guiteras, Anna Sola

**Affiliations:** 1 Department of Ischemia and Inflammation, Experimental Pathology, IIBB-CSIC-IDIBAPS, Barcelona, Spain; 2 Faculty of Medicine, Institute of Biochemistry I, Goethe-University Frankfurt, Frankfurt am Main, Germany; 3 Department of Physiology and Immunology, University of Barcelona, Barcelona, Spain; 4 Department of Internal Medicine, Hospital Clínico Universitario “Lozano Blesa”, Zaragoza, Spain; 5 Department of Experimental Nephrology, (IDIBELL), L’Hospitalet del Llobregat, Pavelló de Govern c/ Feixa Llarga s/n, 08908 Barcelona, Spain; 6 CIBER-BBN, Networking Center on Bioengineering, Biomaterials and Nanomedicine, Zaragoza, Spain

**Keywords:** Cytoskeleton, Physiology

## Abstract

Macrophages show remarkable phenotypic plasticity in response to environmental signals. Although it is generally less considered, cytoskeletal changes in macrophages influence their phenotype, including phagocytosis and secretion of soluble cytokines. Influenza virus NS1A-binding protein (Ivns1abp) belongs to the Kelch family of proteins that play a central role in actin cytoskeleton dynamics by directly associating with F-actin and by protecting against actin derangement. Due to its role in cytoskeleton preservation, the Ivns1abp gene might be a critical regulator of the macrophage phenotype and function under inflammatory conditions. In this study, we determine that the modulation of the Ivns1abp gene in macrophages could modify resistance to macrophages against inflammation and maintain functional phagocytosis. Our results indicate that inflammatory insults inhibit the Ivns1abp gene, whereby phagocytosis is inhibited and the ability of macrophages to induce proliferation and repair of damaged cells is compromised. Furthermore, our results show that inflammatory insults alter the activity of the transcription factor c-myc, a factor which directly modulates the expression of the Ivns1abp gene. In conclusion, this study demonstrates a central role of lvns1abp in promoting and preserving a reparative macrophage phenotype and resistance to this inflammatory environment.

## Introduction

Macrophages exhibit a wide heterogeneity and functional plasticity, which is reflected in their expression of different phenotypes in response to microenvironmental signals. They can be activated conventionally by microbial wall lipopolysaccharides (LPS) and Th1-type cytokines, triggering the activation of a pro-inflammatory phenotype or, alternatively, in the presence of Th-2 type cytokines such as IL-4, IL-13 or IL-10, inducing the alternative phenotype polarization^1^. The biphasic effect of macrophages during damage/repair in tissues is attributed to a macrophage switch towards an alternatively activated phenotype, leading to the suppression of the inflammatory response and the induction of the repair phase^2^. Latter investigations suggest that targeting macrophage activation and phenotype can lead to new therapies in the treatment of acute and chronic diseases such as obesity, insulin resistance or asthma^3,4^. Along the same lines, our group has demonstrated the therapeutic effect of macrophage-cell therapy in different chronic and acute kidney diseases^5–7^.

Apart from the cytokine stimuli, changes in the cell morphology by themselves or as a function of the stiffness of the substrate are also critical for the pro-inflammatory vs. pro-healing regulatory state of macrophages^8,9^. In recent years, it has been demonstrated that modifications in cellular shape influence the transcription of gene programs associated with the macrophages, mechanical signals on transcription factor activity and therefore on genetic pathways associated with phenotype polarization^10–12^. Today, due its complex nature, macrophage polarization remains a subject of much debate and controversy. Some studies using engineered cell culture substrates to control cell shape determined that elongation of macrophages and stabilization of the cytoskeleton promote a pro-healing macrophage phenotype^9,11,13^. Nevertheless, novel biochemical or molecular approaches that expand the knowledge of the underlying mechanisms are urgently needed. The task now is to discover new components that induce genes related to cytoskeleton dynamics and regulation.

The influenza virus NS1A-binding protein (Ivns1abp) gene encodes the actin binding protein, Nd1, which belongs to the Kelch family of proteins. These proteins play an important role in the organization of actin cytoskeleton dynamics by stabilizing actin filaments through their association with F-actin mediated by Kelch repeats^14^. Both Nd1-S and L cross-link actin filaments, protect against the actin derangement, and play a critical role in fundamental cellular functions such as cell division, cell locomotion and phagocytosis. Indeed, it has been reported that overexpression of Nd1-S modifies cell cycle progression in fibroblasts^15^. These features make Ivns1abp an important regulator of cytoskeleton stability and, consequently, a potential critical regulator of the macrophage phenotype.

Ivns1abp gene promoter is regulated by c-myc^16^, among others,. In turn, c-myc is modified by a variety of downstream signalling pathways of inflammatory cytokines^17–19^, and is also involved in the induction of a large set of genes during alternative macrophage activation, by way of either direct or indirect interaction^20^.

Since, on the one hand, the Ivns1abp gene takes on an important role in both the coordination of actin cytoskeleton dynamics as well as cell shape and, on the other hand, modifications of the macrophage cytoskeleton can regulate their pro-inflammatory state versus the pro-healing active state, in this study we investigated the contribution of the Ivns1abp gene in the control of the actin cytoskeleton of macrophages during inflammation and the role of this gene in macrophage polarization. We also explored the role of transcription factor c-myc in this context. We further tested the potential therapeutic effect as cell therapy against renal I/R injury.

## Material and methods

### Animals

All procedures were approved and carried out under the supervision of our institution’s Ethics Committee for Animal Experimentation (Comitè d’Ética d’Experimentació Animal, CEEA) and in accordance with the approved European Union guidelines for the handling and care of laboratory animals. We used Sprague Dawley rats (225–250 g) (Charles River, France) for cell therapy studies involving ischemia/reperfusion injury. Animals were given free access to water and a standard laboratory chow diet.* In vivo experimental groups (n = 5 per group):* Animals were anesthetized with isoflurane and placed in a supine position with body temperature maintained at 37ºC. The abdominal area was covered to minimize dehydration. After laparotomy and dissection of renal pedicles, bilateral ischemia was induced by occluding the renal pedicles with an atraumatic microvascular clamp for 45 min. The clamps were removed during reperfusion. Sham: control group without infusion of macrophages; Sham + Mϕ-Ivns1abp: control group with adoptive transfer of *ex-vivo*–modified macrophages overexpressing Ivns1abp; ischemia/ reperfusion (I/R): 45 min of bilateral ischemia followed by 24 h of reperfusion; I/R + Mϕ-Ivns1abp: I/R group with adoptive transfer of ex vivo–modified macrophages overexpressing Ivns1abp; I/R + Mϕ-βgal: I/R group injected with β-gal macrophages. At 24 h of reperfusion, blood was collected for creatinine and blood urea nitrogen analysed. In addition, kidneys were harvested and snap-frozen at -80ºC, set in paraformaldehyde, or embedded in an optical coherence tomograph and frozen in liquid nitrogen. For the adoptive transfer, macrophages were smoothly transferred to a culture tube, and maintained in PBS until injection into the animal. We injected the cells (10 × 10^6^ cells per rat, via direct intravenous puncture in the inferior vena cava) 1 h after reperfusion induction as previously performed by our group^5^.

### Primary macrophage generation

Rat bone marrow-derived macrophages (BMDM) were isolated from Sprague Dawley rats (Charles River, Barcelona, Spain) by aspiration of the femur. They were then re-suspended in DMEM, supplemented with 10% FCS, 10 ng/ml GM-CSF (Peprotech, Hamburg, Germany), 100 U/ml penicillin, and 100 µg/ml streptomycin, and kept in non-adherent culture flasks for 7 days. Macrophages were subsequently isolated by adherence and purity was determined by ED1 and CD11b expression.

### NRK-52e culture

The renal epithelial cells NRK-52e (European Collection of Cell Culture, Salisbury, UK) were cultured in Dulbecco’s modified Eagle Medium (DMEM) with high glucose, 15 mM Hepes and L-glutamine, supplemented with 100 U/ml of penicillin, 100 µg/ml of streptomycin, and 10% FCS. Cells were kept in a humidified atmosphere with 5% CO_2_ in air at 37 °C and were passaged every second day using 1 mM EDTA/0.025% trypsin (Gibco). Cell cycle arrest was performed as indicated previously by our group^21^. As described, 50 µM colchicine (Sigma, Madrid, Spain) was added to NRK-52e cells for 24 h to decrease proliferation without compromising viability. Colchicine was removed, fresh medium was added and cell viability was checked. Individual stimuli were applied for another 24 h.

### Renal epithelial cell/macrophage co-culture

Co-culture conditions of NRK-52e cells and primary macrophages at a ratio of 1:1.5 was performed for 24 h in 6-well plates using transwell inserts (Corning, Madrid, Spain) to ensure spatial separation. NRK 52e cells (bottom) were treated with colchicine for 24 h and washed twice with PBS prior to co-culture with primary bone-marrow-derived macrophages (BMDM) seeded into the insert of the transwell system^21^. At the end of the culture, cells and supernatant were processed separately for further determinations.

### Preparation of macrophage conditioned media

For the generation of conditioned media, cells were serum-starved for 24 h. Primary macrophages were then transfected either with siRNA to knockdown Ivns1abp (shIvns1abp) or with non-targeting shRNA. After removing the supernatant, macrophages were washed twice with PBS, and incubated for another 24 h with DMEM without serum. This supernatant was termed conditioned medium (CM) and subsequently used in further experiments as indicated. CM was harvested by way of centrifugation (13.000 × *g*, 10 min) and filtered through 0.2 μm pore filters (Millipore, Madrid, Spain) to remove large particles.

### Adenoviral transfection

Adenoviral vectors were used to transduce BMDM ex vivo. shRNA transfection as well as adenoviral vectors carrying cDNA encoding recombinant Ivns1abp, β-galactosidase or GFP was performed to the manufacturer’s recommended protocol EBioFocus DPI BV, Leiden, Netherland for shRNA). Adenoviral vectors for β-galactosidase (Adβ-gal) were kindly provided by Dr David Kluth and Dr Jeremy Hughes (MRC Centre for Inflammation Research, The Queen's Medical Research Institute, Edinburgh, UK). The adenoviral vector carrying the cDNA encoding recombinant Ivns1abp was elaborated, amplified, and purified by ViraQuest, Inc (North Liberty, IA, USA). We used transduction medium containing (DMEM (high glucose)/F12 + GlutaMax 2% FBS), 1.6 µl of poly-l-lysine and 800 μl of virus according to its optimum dose (MOI 100 for sh-Ivns1abp and MOI 250 for Ivns1abp, β-gal, and GFP). Cells were incubated for 24 h at 37ºC in a CO_2_ incubator. Quantification of ivns1abp expression was performed by real time qRT-PCR at 24 h post transfection.

### In vitro experimental groups

*For BMDMs;* Resting Mϕ: Untreated BMDMs; Mϕ sh-Ivns1abp: BMDMs transfected with a vector that silences Ivns1abp expression; Mϕ non-targeting shRNA; shRNA targeting eGFP; Mϕ + Ivns1abp: vector over-expressing Ivns1abp gene; Mϕ + cytokines: BMDMs treated with cytokine cocktail (60 ng/ml TNF-α, 100U/ml IFN-γ); Mϕ + Ivns1abp + cytokines: Ivns1abp over-expression and treatment for 24 h with cytokine cocktail; Mϕ + βgal: Transduction with adenoviral control vector β-galactosidase; Mϕ + F4: treatment for 24 h with 100 μm of the c-myc inhibitor 10058-F4. *For epithelial cells;* Control: untreated cells; NRK: Epithelial cells were treated with 50 μM of colchicine for 24 h; NRK + Mϕ: cells treated with colchicine were co-cultured with unstimulated BMDM for 24 h; NRK + Mϕ non-targeting shRNA or with a vector that silences the expression of Ivns1abp gene (NRK + Mϕ sh-Ivns1abp).

### Scratch wound assay

For scratch wound assays, 1 × 10^5^ NRK-52e cells were seeded in 24-well culture plates until 80% confluence was obtained. After 24 h, cells were starved for an additional 16 h before the scratch was carried out with a small pipette tip. Detached cells were removed by washing with PBS. Stimulation was performed with conditioned medium for 24 h.

### Phagocytosis assay

In order to examine the phagocytic function of BMDM cells, phagocytosis assay tests were performed. BMDM cells after being differentiated for 7 days and seeded to 6-well plates, were stimulated for 24 h, either with DMSO (1:1,000) or with TNF-a (60 ng/ml, 3 μl/ well from stock 20 μg/ml), IFN-y (100 U/ml, 1 μl/ Stock 100 U/μl) or c-myc Inhibitor (1:1,000, 100 μM, 1 μl/well). The conditioned medium was removed and FCS free media with 10 μM CMRA was added and incubated with the cells for 15–45 min. Subsequently, the working dye solution was replaced with fresh, pre-warmed medium and cells were incubated for another 30 min at 37 °C. Fluorescent carboxylate beads (2 μm diameter, Invitrogen, Carlsbad, CA) were incubated with the cells for a maximum of 120 min. Samples, after the different time periods of contact with fluorescent carboxylate, were washed extensively with PBS and 500 μl of accutase per well for 5 min in order to detach the cells for flow cytometry analysis. Samples were placed in FACS tubes containing 1–2 ml of PBS to stop accutase reaction. 500 μl of the sample was used for RNA analysis while the remainder was centrifuged (5 min, 500 g, 4 °C) to remove PBS and accutase and blocked for 15 min using Fc Block MIX (0.5 μl FcBlock in PBS). After blocking, F4/80 Antibody PBS mix (1 μl/ sample in 100 μl PBS) was added and cells were analyzed by flow cytometry. For microscope visualization, cells were washed with PBS and fixed with 3.7% formaldehyde in PBS for 15 min at room temperature. Cells were washed with PBS and incubated with Hoechst Farbstoff (0.2 μg/ml 1:5,000) for 7 min. After washing with PBS and water, samples were mounted with vectashield and viewed using a Leica TCS NT laser microscope (Leica Microsystems, Wetzlar, Germany).

### Phalloidin staining

For the in vitro experiments, cells were fixed in 3.7% paraformaldehyde for 10 min, washed with PBS, permeabilized with acetone for 5 min on ice, and blocked with goat serum (Sigma, Madrid, Spain) for 1 h at room temperature. Cells were washed twice with PBS and incubated with Alexa Fluor 568 phalloidin staining (Invitrogen) 0.5 Units/100 μl l in 1% PBS/BSA buffer for 30 min at room temperature. After washing with PBS, samples were incubated with DAPI solution (0.2 μg/mL) (Sigma-Aldrich) for 5 min and washed three times with PBS. The preparation was mounted with mowiol (Calbiochem, Madrid, Spain) and cells were viewed using a Leica TCS NT laser microscope (Leica Microsystems, Wetzlar, Germany). In the case of kidney tissue, one-half of the kidney was embedded in OCT and frozen in liquid nitrogen without prior fixation. Five-micrometer cryosections were fixed in 4% buffered formaldehyde for 10 min and then permeabilized with PBS containing 0.1% Triton X-100 and 1% BSA for 30 min. For actin visualization, the slides were incubated with Alexa Fluor 568-phalloidin (dilution, 1:40, Molecular Probes, Eugene, OR) in PBS with 1% BSA for 30 min. Slides were washed three times for 15 min with PBS and finally mounted using mowiol. Confocal images were taken with a Leica TCS NT laser microscope (Leica Microsystems, Wetzlar, Germany) equipped with a 100 oil-immersion objective as previously described^22^.

### SDS-PAGE and Western blot analysis

For Western blot analysis, cells were scraped off, centrifuged, resuspended in lysis buffer (Urea 7 M, Thiourea 2 M, Chaps 4%, Tris 40 mM, PMSF, DTT), and sonicated for 1 min. Subsequently, protein concentrations were determined with the Bradford method using a commercial kit from BioRad. Equal amounts of protein were resolved on 10%-SDS polyacrylamide gels and blotted onto nitrocellulose membranes. Unspecific binding was blocked with 5% milk in 0.6% Tween-TBS (TTBS) for 1 h. Primary antibody was added in TTBS/5% dry milk and incubated overnight at 4 °C. For protein detection, blots were incubated with the secondary antibody in TTBS/5% dry milk, followed by ECL-detection of specific proteins using the Quantity One imaging system (Bio-Rad). Equal loading of protein was determined by visual detection by incubating the membranes with Ponceau. The sample treatment in the actin Western blot was performed to differentiate the F- and G-actin forms. The samples were homogenised in PHEM buffer (60 mM PIPES, 25 mM HEPES, 10 mM EGTA, and 2 mM MgCl2, pH 6.9, containing 0.1% Triton X-100, 0.5 mM PMSF, and 0.1 mM DTT). The homogenate was centrifuged at 48,000 g during 5 min at 4 °C. The insoluble fraction (cytoskeleton associated) was used for Western blot analysis^13^.

### Total RNA extraction, cDNA synthesis and real time PCR

Total RNA was isolated using the RNeasy mini kit following the manufacturer’s protocol (Qiagen, Barcelona, Spain). Thereafter, RNA was eluted in 50 μl of RNase-free water. RNA concentrations were calculated from A260 determinations using a Nanodrop ND-1000 (NanoDrop Technologies, Wilmington, DE, USA. Then, obtained RNA was reverse transcribed by using an iScript cDNA Synthesis Kit (Bio-Rad) in a final volume of 20 μl. Quantitative RT-PCRs were performed on a Bio-Rad iCycler iQ Real-Time-PCR detection system using either SYBR Green RT-PCR detection Kit (Bio-Rad, Madrid, Spain) or pre-validated TaqMan probes (Applied Biosystems, Madrid, Spain) according to manufacturer’s instructions. For the design of the primers internet sources such as NCBI, Primer 3 and Operon were used. Table 1 shows selected primers used in the study.

**Table 1:** Primer sequences used in this study.

Gene (Accession number)	Source	Primer sequence
GAPDH (NM_ 008084)	Invitrogen	Forward: 5′-TGA-AGC-AGG-CAT-CTG-AGG-C-3′ Reverse. 5′-CGA-AGG-TGG-AAG-AGT-GGG-AG-3′
Ki-67 (NM_001081117)	Invitrogen	Forward: 5′-CAG-TAC-TCG-GAA-TGC-AGC-AA -3′ Reverse: 5′-CAG-TCT-TCA-GGG-GCT-CTG-TC-3′
PCNA (NM_011045)	Invitrogen	Forward: 5′-AAT-GGG-GTG-AAG-TTT-TCT-GC-3 Reverse: 5′-CAG-TGG-AGT-GGC-TTT-TGT-GA-3′
Mannose Receptor (NM_008625)	Invitrogen	Forward: 5′-CAG-GTG-GCT-TAT-GGG-ATG-TT-3′ Reverse: 5′-GAG-TTG-TTG-TGG-GCT-CTG-GT-3′
TNF-α (NM_013693.3)	Invitrogen	Forward: 5′-GAA-CTG-GCA-GAA-GAG-GCA-CT-3′ Reverse: 5′-GGT-CTG-GGC-CAT-AGA-ACT-GA-3′
iNOS (NM_010927.3)	Qiagen	QuantiTect Primer Assay for SybrGreen detection
IL-10 (NM_010548)	Qiagen	QuantiTect Primer Assay for SybrGreen detection
Ivns1abp (NM_001039511.1)	Applied biosystems	TaqMan Gene Expression Assay
c-myc (AFO76523)	Applied biosystems	TaqMan Gene Expression Assay
GAPDH (NM_008084.2)	Applied biosystems	TaqMan Gene Expression Assay
Rn_PCNA (NM_022381)	Invitrogen	Forward: 5′-AGG-ACG-GGG-TGA-AGT-TTT-CT-3′ Reverse: 5′-CAG-TGG-AGT-GGC-TTT-TGT-GA-3′
Rn_Ki-67 (XM_00105622)	Invitrogen	Forward: 5′-AGA-CGT-GAC-TGG-TTC-CCA-AC-3′ Reverse: 5′-ACT-GCT-TCC-CGA-GAA-CTG-AA-3′
Rn_GAPDH (NM_017008)	Invitrogen	Forward: 5′-CCG-CCA-ATG-TAT-CCG-TTG-TG-3′ Reverse: 5′-TAG-CCC-AGG-ATG-CCC-TTT-AGT-3′

### Statistical analysis

All data are reported as mean ± SE. Group means were compared with either the Students *t* test or ANOVA for parametric values, *P* ≤ 0.05 was considered to be statistically significant.

## Results

### Modulation of Ivns1abp gene in macrophages increases the resistance against inflammation and cytoskeleton derangement provoked by cytokines

To explore the impact of the Ivns1abp gene on the macrophage cytoskeleton, inflammatory state and reparative capacity, we used a specific adenovirus as the RNA interference-based gene silencing technology to knock out Ivns1abp expression.

**Figure 1:**
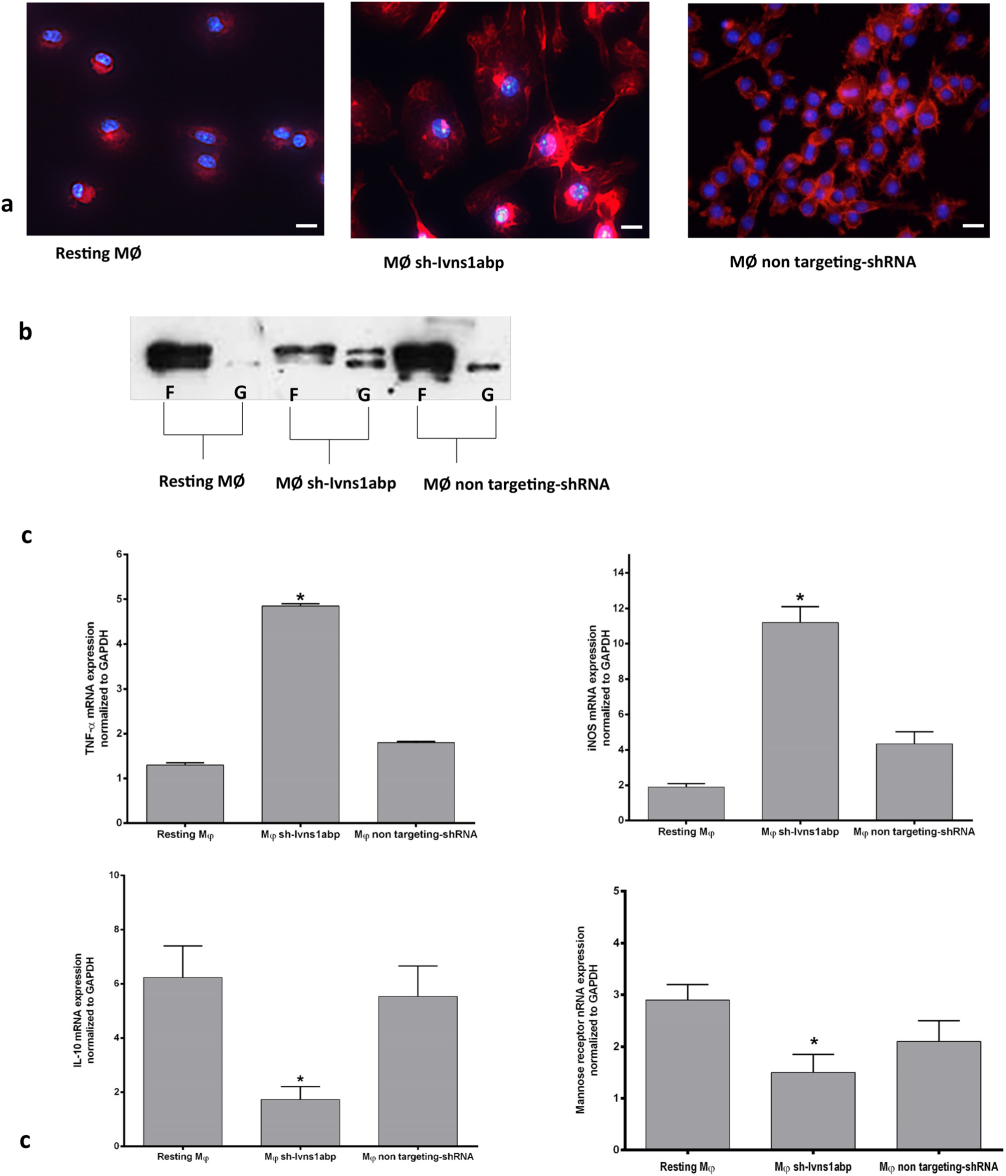
Representative images of (**a**) phalloidin staining of non-treated BMDM, BMDM silenced for the Ivns1abp gene or transduced with non-targeting shRNA. Knockdown of Ivns1abp causes loss of the stress fibers in the cytoskeleton and the consequent formation of actin clusters. Original magnification 40×. Bar = 25 μm (**b**) Western blot for the cytoskeleton-associated fraction of actin corresponding to F-actin (F, filamentous) versus G-actin (G, globular) (cropped blot). (**c**) Expression of pro and anti-inflammatory markers in the described groups. The down regulation of gene expression induces macrophages to adopt a pro-inflammatory state. Data is represented as mean ± SEM. ∗*p* < 0.05 versus Resting Mφ; n = 5.

**Figure 2:**
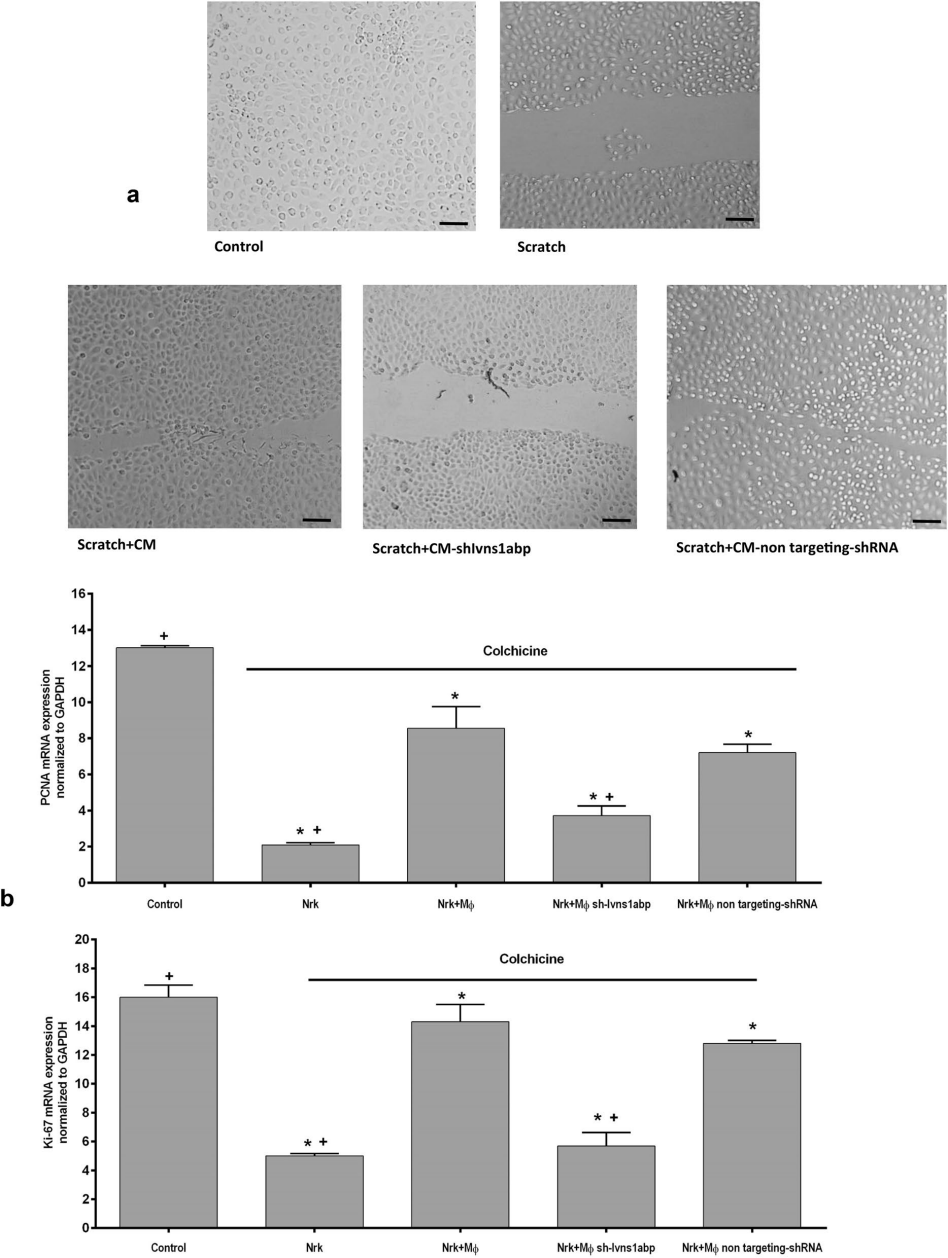
(**a**) Representative pictures of the ability of the macrophage-conditioned medium promote migration and repair of the epithelial cells after a mechanical damage. The different conditioned medium was exposed to the epithelial cells during 24 h after the scratch Representative images of 5 experiments. Original magnification 20×. Bar = 75 μm (**b**) Expression of the proliferation markers PCNA and Ki-67 of NRK-52e renal epithelial cells treated with colchicine to arrest cell cycle and after co-culture with untreated or Ivns1abp-silenced macrophages. Data is represented as mean ± SEM. ∗*p* < 0.05 versus NRK + Col + Mφ; n = 5.

Figures 1 and 2 show the Ivns1abp-specific down-regulation effects in macrophages. The organization of cytoskeletal F-actin was visualized by immunofluorescence and further determined by F-actin and G-actin fractions. The images show a round shape in resting cells with bright fluorescent staining and homogenous distribution of the staining along the cytoplasm. Down-regulation of Ivn1sabp induced irregular shaped cells, and evident disruption of actin filaments with brightness dissipation and the presence of F-actin fibres in clusters showing a non-homogeneous distribution of the fluorescence along the cytoplasm (Fig. 1a). The observed changes in actin filament were confirmed by WB analysis. Upon Ivns1abp knockdown a loss of F-actin and a gain of G-actin fraction was observed (Fig. 1b). Since cell shape is related to macrophage phenotype, we analysed the transcription of pro-inflammatory and anti-inflammatory markers. iNOS and TNF-α levels increased, whereas the anti-inflammatory IL-10 and mannose receptor significantly decreased (Fig. 1c). Thus, the results indicate that the Ivns1abp gene is implicated in morphology, shape as well as phenotype activation in macrophages. The non-targeting shRNA group remained unaffected. The additional data 1a shows the expression of Ivns1abp gene, demonstrating the efficiency in downregulation by expressing only 12–15% of the Ivns1abp gene compared to the level of expression of untreated macrophages.

With two different tests, we then analysed the macrophage cytoskeleton modifications based on repair function. First, we tested the reparative capacity of macrophage supernatants using a cellular scratch model (Fig. 2a), and second, we determined the ability to induce proliferation by enhancing Ki-67 and PCNA mRNA expression in a co-culture with renal epithelial cells (Fig. 2b). In the scratch test, we added different macrophage supernatants to damaged (scratched) renal epithelial cells for 24 h and we observed that the supernatant of the Ivns1abp-deficient macrophages lost its ability to induce repair compared to the that of the standard control macrophage or non-targeting shRNA macrophage supernatant capacity. In the case of the co-culture test, epithelial cells were pre-treated overnight with colchicine to provoke cell cycle arrest, a model that our group had already established beforehand^21^. Epithelial cells were washed after colchicine exposure and were either left untreated (control) or were co-cultured in a transwell system with macrophages for an additional 24 h. Data depicts that colchicine treatment by itself considerably decreased the mRNA expression of the proliferative markers PCNA (Fig. 2b, upper panel) and Ki-67 (Fig. 2b, lower panel) of the epithelial cells compared to control cells. Co-culture with control macrophages or macrophages transfected with non-targeting shRNA positively re-induces the expression of these markers, demonstrating the capacity of macrophages in the re-establishment of the epithelial cell-cycle. However, co-culture with macrophages transduced with sh-Ivns1abp did not promote the activation of the proliferative markers, indicating that the reparative and proliferative capacity of macrophages in epithelial cells is dependent on, at least in part, Ivns1abp gene activation.

**Figure 3:**
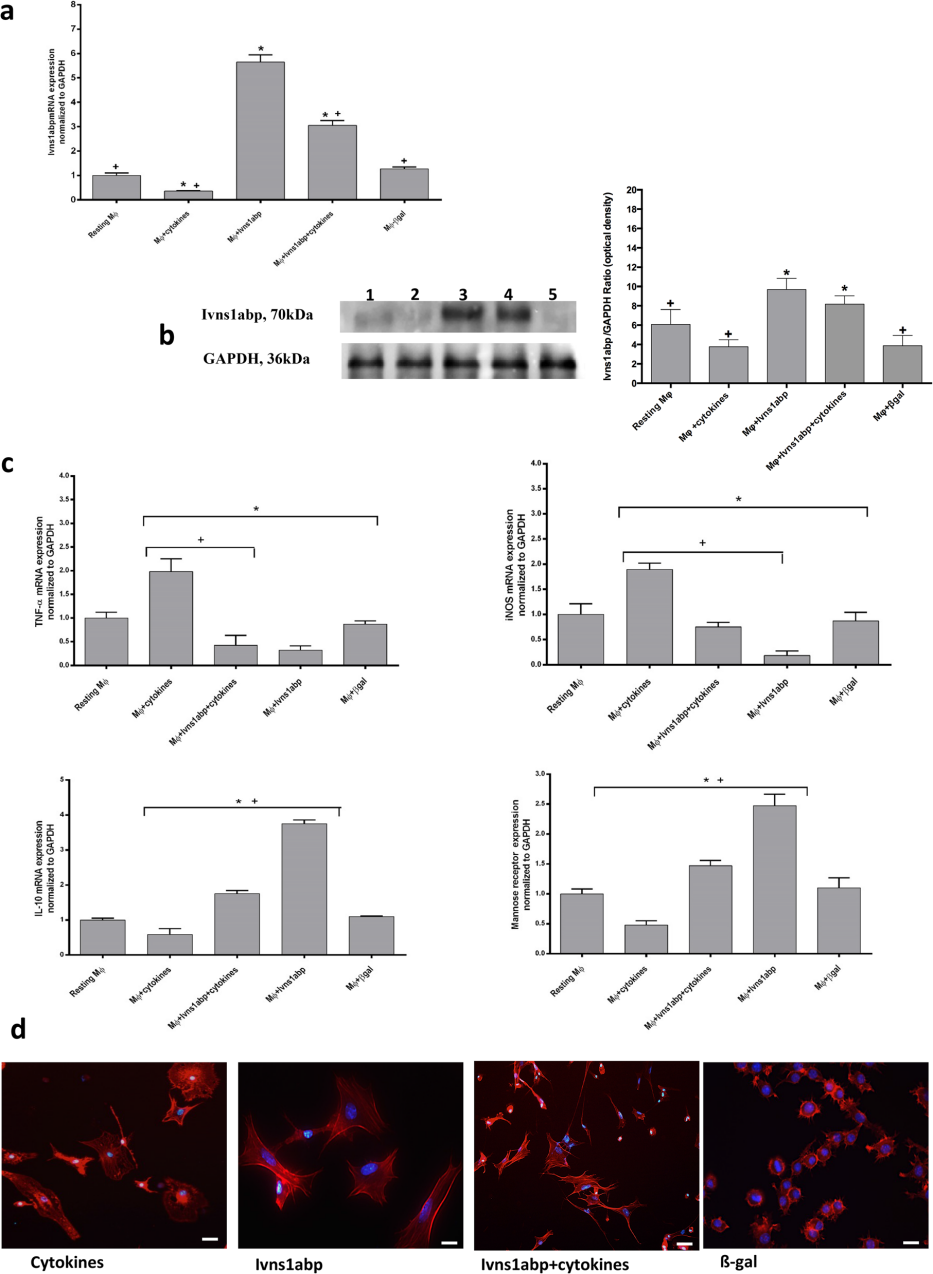
(**a**) Expression of Ivns1ap gene and (**b**) and western blot for Ivns1abp and GAPDH on macrophages. Protein bands are at the expected size of 70 KDa for Ivns1abp and 36 kDa for GAPDH (cropped blot). Right panel, densitometric analysis ratio of protein bands. (**c**) Expression of pro-inflammatory (TNF-α, iNOS) and anti-inflammatory markers (IL-10 and Mannose Receptor) in macrophages transfected with Ivns1abp and treated with pro-inflammatory stimulus (cytokine cocktail). Data is represented as mean ± SEM n = 5. ∗*p* < 0.05 versus Resting Mφ; + *p*< 0.05 versus Mφ + Ivns1abp (**d**) Representative pictures of phalloidin staining of macrophages treated with cytokines and macrophages over-expressing Ivns1abp with or without cytokines treatment. Original magnification 40×. Bar = 25 μm.

In order to study whether this gene plays a role in macrophage phenotype and the response to inflammation, we generated Ivns1abp-overexpressing macrophages and treated them with pro-inflammatory cocktail (TNF-α and IFN-γ), widely described experimentally as pro-inflammatory stimuli^23–25^. Pro-inflammatory cytokines showed the ability to modulate the expression of the Ivns1abp gene as well as the protein in both primary macrophages and in those with Ivns1abp overexpression. (Fig. 3a,b). Some experimental variations may appear between primary cells, but the levels of expression of Ivns1abp protein in resting cells and b-gal control cells are low. Although the modulatory action, we tested whether the up-regulation of Ivns1abp induced an anti-inflammatory phenotype, cytoskeleton preservation and whether this provided resistance to inflammatory cytokines. Ivns1abp-upregulated macrophages responded to cytokine stimulation by maintaining the expression of high levels of anti-inflammatory markers (mannose receptor and IL-10) and low levels of pro-inflammatory markers (TNF-α and iNOS) showing resistance to the inflammatory insult (Fig. 3c). The morphology and shape of the cells are displayed in Fig. 3d; macrophages transfected with Ivns1abp modifies the round shape and the F-actin distribution found in resting cells. The morphology became elongated, flattened and more spread out. F-actin appears organized and visualized into stress fibers covering the whole cytoplasm. By contrast, cytokine administered to resting cells promoted a less elongated shape, a generally lower intensity of the brightness of the staining, and stress fibers were observed to be discontinuous and disorganized. Intense clusters also appeared around the nucleus. The effect of cytokine treatment is attenuated when Ivns-1abp is upregulated and, although the intensity of the stain decreases and the morphology is modified to some degree, the cells preserve the elongated morphology and the continuous stress fibers along the cytoplasm still remain. These observations indicate that the Ivns1abp over-expression in macrophages mitigates the effect of cytokines and promotes anti-inflammatory status.

The efficiency of the transduction is showed in the additional data 1b.

**Figure 4:**
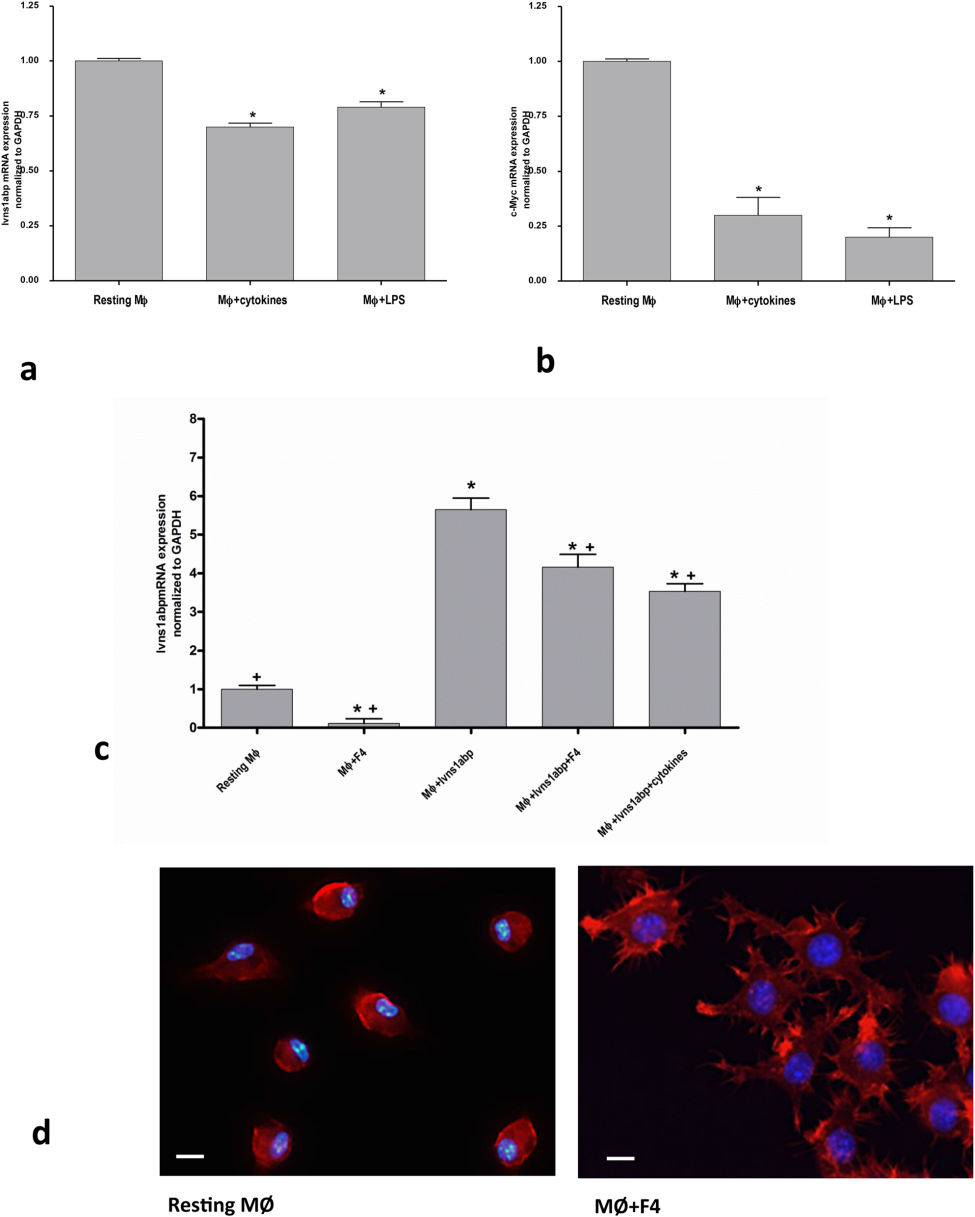
Ivns1abp (**a**) and c-myc expression (**b**) in resting macrophages or treated with pro-inflammatory stimulus cytokines cocktail (TNF-α and IFN-γ) or LPS. Data is represented as mean ± SEM n = 5. ∗ p < 0.05 versus Resting Mφ (**c**) Expression of Ivns1abp in macrophages treated with or without cytokines and c-myc inhibitor. Data is represented as mean ± SEM. ∗*p* < 0.05 versus Resting Mφ; +*p* < 0.05 versus Mφ + Ivns1abp n = 5 (**d**) Representative images of phalloidin staining of resting macrophages and macrophages treated with c-myc inhibitor. Original magnification 40×. Bar = 25 μm.

In order to confirm that other inflammatory insults have a similar effect on the Ivns1abp transcription, we used LPS as a pro-inflammatory inducer. Similarly, both the cytokine cocktail and LPS caused the downregulation of Ivns1abp (Fig. 4a). Interestingly, the inhibition of its transcription factor c-myc, was also clear (Fig. 4b). The relation between pro-inflammatory stimulus and c-myc regulation in macrophages is well described^26,27^. In order to explore the relationship between Ivns1abp and c-myc in our settings, we used 10058-F4, a c-myc inhibitor that prevents transactivation of c-myc target gene expression. Figure 4c shows that macrophages treated with c-myc inhibitor, present a significant decrease in the levels of Ivns1abp expression compared to the those of the control group, indicating the ability of c-myc to directly modulate Ivns1abp expression. This expression is also decreased in macrophages over-expressing Ivns1abp and treated with cytokines or with c-myc inhibitor. The effect of the inhibitor on Ivns1abp protein expression is shown and quantified in the additional data 1c.

To test if the c-myc-dependent modulation of Ivns1abp induces direct cytoskeleton destabilization, we performed phalloidin staining in control macrophages and macrophages treated with 10058-F4 (Fig. 4d). The representative pictures show that control cells displayed a round shape and normally-distributed actin. However, groups treated with c-myc inhibitor display a change in the morphology and shape of the cells. The F-actin stain was not homogenously distributed and some more intense staining appeared as clusters with tightly packed texture, but no long stress fibers were detected.

Altogether, these results demonstrate that cytoskeleton disorganization induced by proinfammatory stimulusis modulates ivns1abp gen.

### c-myc and Ivns1abp modulate macrophage resistance to cytokines and induce functional phagocytic response

**Figure 5:**
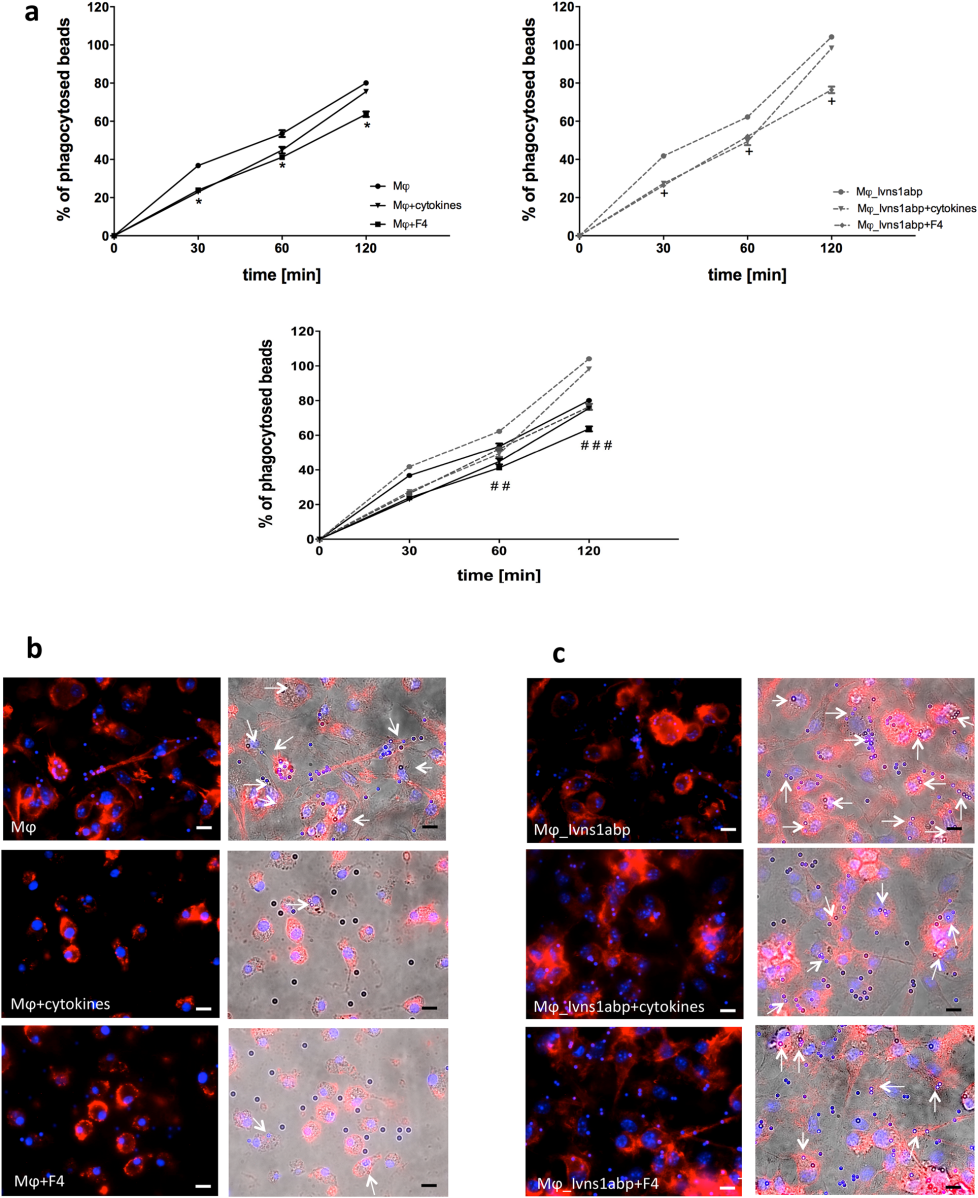
(**a**) Kinetics of the phagocytosis assay at different time points using Carboxylate-Modified Microspheres in non-transfected macrophages (left) in macrophages overexpressing Ivns1abp (right) and all groups merged in one graph (down). Data is represented as mean ± SEM. n = 5. *∗*p < 0.05 versus Mφ at each time point, +*p* < 0.05 versus Mφ_Ivnsapb at each time point, ♯♯♯*p* < 0.05 significant in all overexpressed versus non-overexpressed groups at this time point. ♯♯*p* < 0.05 Mφ and Mφ + F4 versus corresponding over-expressed groups in the time point. Representative fluorescent images (right) and overlay with optical view (left) of the phagocytosis assay at 60 min in (**b**) non-overexpressed groups and (**c**) and in over-expressed groups. White arrows point the phagocytic beads. Un-phagocyted beads are detected by larger size and different colour. Original magnification 40×. Bar = 25 μm.

In order to find out if the changes in the cytoskeleton also affected the phagocytic capacity, we performed a phagocytosis assay using FluoSpheres Carboxylate-Modified Microspheres, 2.0 µm. Macrophages were stained with F4/80-PE-Cy7 antibody and were analysed by flow cytometry at different time points. Figure 5a shows the phagocytosis results in the different groups with or without Ivns1abp overexpression. The results of the kinetics show that both the cytokines and the c-myc inhibitor, decrease the phagocytic capacity compared to their respective controls. It should also be noted that the over-expression of Ivns1abp increases the phagocytic capacity of macrophages despite the presence of cytokines or c-myc inhibitor. Figure 5b,c shows representative images of the phagocytosis during the assay. The phagocytosed beads could be detected in the cytoplasm of the cells by the change in size and colour. The white arrows indicate the phagocytic beads that are displayed small and bright inside the cell. On the other hand, the unphagocytosed beads can be identified by their larger size and by their intense blue colour outside the cells. The image demonstrates that overexpression of Ivns1abp increases the quantity of the phagocytized beads independently of the treatment but from them the phagocytosis is especially active in resting macrophages while pro-inflammatory cytokines or c-myc inhibition decrease it. These results indicate that both factors act on the reduction of the phagocytic capacity in macrophages.

### Over-expressing Ivns1abp macrophage-cell therapy preserves the renal cytoskeleton and improves renal function against I/R insult

**Figure 6:**
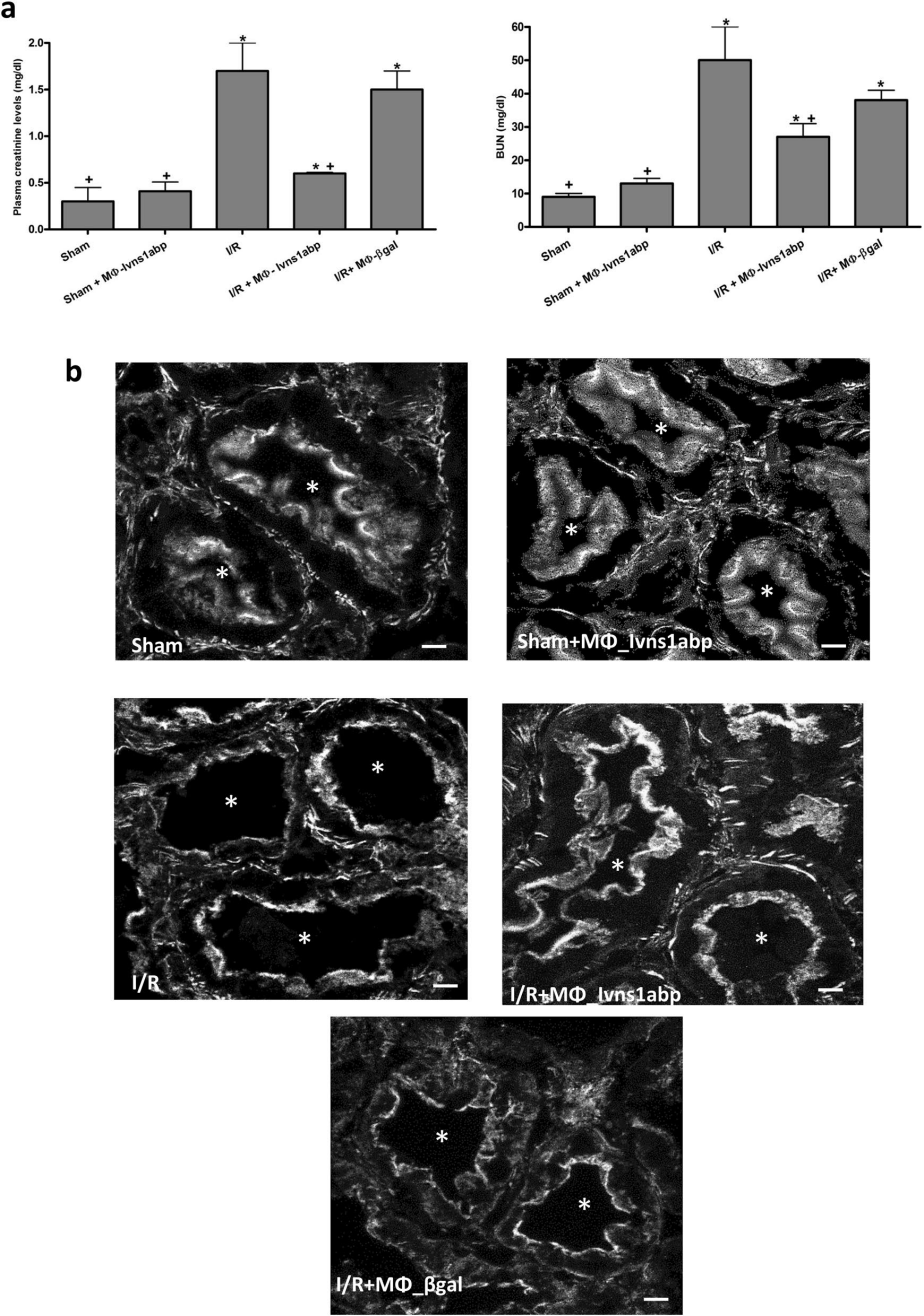
(**a**) Creatinine and blood urea nitrogen (BUN) in plasma of Sham, IR groups and adoptive transfer of either macrophage transduced with β-gal or Ivns1abp. Data is represented as mean ± SEM n = 5. ∗*p* < 0.05 versus Sham; +*p* < 0.05 versus I/R. (**b**) Representative images of F-actin visualization by phalloidin of proximal renal tubules from cortical kidney sections and analyzed by confocal microscopy in Sham and I/R animals with or without cell therapy of macrophages overexpressing Ivns1abp or macrophages transduced with β-gal adenovirus. The asterisk indicates the lumen of the tubules. Original magnification 60×. Bar = 10 μm.

We already demonstrated that cell therapy with modified macrophages ameliorate kidney damage in different experimental renal pathologies by decreasing the inflammatory scenario^5–7^. Given the properties of Ivns1abp-macrophages in actin cytoskeleton stability and the resistance against inflammatory insults, we therefore investigated whether cell therapy with these modified macrophages preserve kidney function and epithelial integrity against the inflammatory damage associated with I/R injury. Figure 6 shows renal function and renal tubule integrity after I/R. Blood urea nitrogen and creatinine (Fig. 6a) at 24 h of reperfusion are increased, thus indicating a decrease in renal function. Adoptive cell transfer of Ivns1abp-over-expressing macrophages to I/R prevented renal dysfunction measured by serum creatinine and BUN compared to the I/R group. In contrast, the administration of the control βgal-adenovirus transduced macrophages did not show significant amelioration in these parameters compared to the I/R group. Thus, BMDMs over-expressing Ivns1abp were highly protective against the loss of kidney function. Cytoskeleton images of renal tubules are shown in Fig. 6b. Phalloidin staining in Sham groups show a clear delimitation and integrity in the epithelial tubule and the villi, which are clearly visualized in the lumen of the tubules. We observed similar images of cytoskeleton pattern in the animals with adoptive transfer of up-regulated macrophages. However, in I/R group, the villi are less apparent, suggesting that they are lost. These results indicate the capability of this cell therapy to prevent actin cytoskeletal alterations.

**Figure 7:**
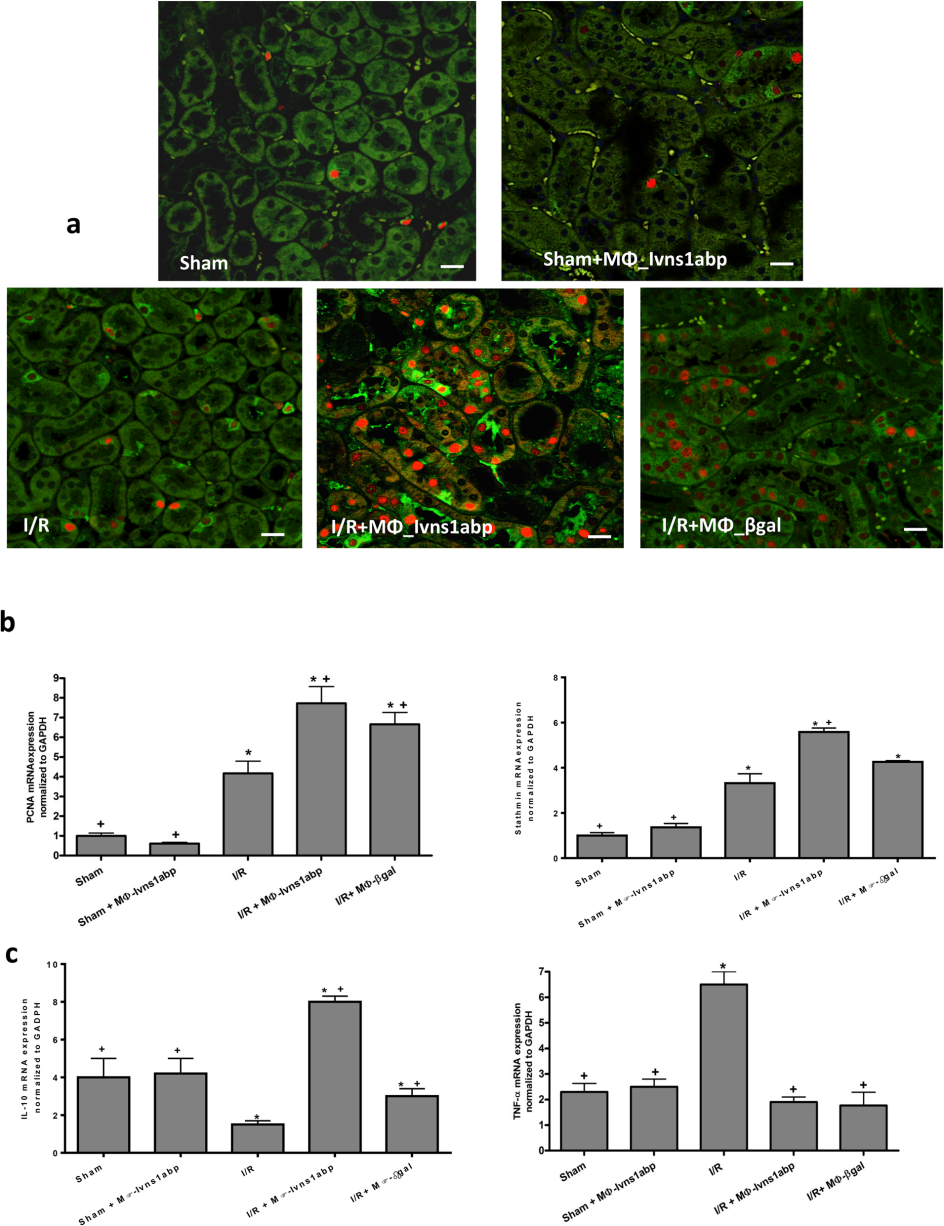
(**a**) Immunostaining of stathmin (green) and PCNA (red) expression in renal tissue of Sham and I/R animals with or without cell therapy of macrophages overexpressing Ivns1abp or macrophages transduced with β-gal adenovirus. Ki-67 and PCNA Original magnification 20×. Bar = 75 μm. mRNA expression tested via qRT-PCR in total renal tissue homogenates of (**b**) PCNA and stathmine and (**c**) IL-10 and TNF-α. qRT-PCR data was normalized to the housekeeping gene GAPDH and expressed as arbitrary units of relative expression. Data is represented as means ± S.E.M. n = 5; *p < 0.05 versus Sham; + *p* < 0.05 versus I/R.

Lastly, we verified whether the observed tissue preservation was also due to inducing greater repair and proliferation of the epithelial tubules. To this aim we measured the specific markers stathmin and proliferation cellular nuclear antigen (PCNA) protein levels by immunofluorescence and the mRNA expression by qRT-PCR. Stathmin (green) staining and proliferation of the cellular nuclear antigen (PCNA) (red) (Fig. 7a) as well as the mRNA levels (Fig. 7b) reflects a clear positive increase of the proliferative markers in the tubule cells especially in the animals treated with Ivns1abp-macrophage cell therapy, indicating the ability to potentiate proliferation and repair. The repair after the I/R injury of the Ivns1abp-macrophages is evidenced by the decrease in tissue inflammation. This is reflected by the increase of the production of the anti-inflammatory cytokine IL-10 and the non-increase in TNF-α expression (Fig. 7c) in these animals. Thus, these results showed the ability of BMDMs overexpressing Ivns1abp in bolstering repair in the kidney against I/R.

## Discussion

The alternative phenotype of macrophages is crucial for stimulating tissue repair and regeneration. But this phenotype is strongly influenced by cytokines and by the inflammatory environment, which diminishes or even eliminates the capacity of repair. Therefore, factors that are capable of eluding cytokine attack and/or promoting resistance to cytokines or to pro-inflammatory insults should be considered in a repair scenario. In this study, we focused on the modulation of the Ivns1abp gene in macrophages, which was revealed as an important target due to its ability to induce resistance to inflammation. Ivns1abp belongs to the Kelch family^14^ and although there are not many studies focusing specifically on the function, they indicate that it is multifunctional, since it is involved in signal transduction^28^, splicing and stability of proteins^29,30^ and stabilization of actin filaments^14^. Our results show that down-regulation of Ivns1bp impacts the actin cytoskeleton of macrophages, directly changing its shape and morphology and promotes its pro-inflammatory state.

The relationship between macrophage morphology and phenotype has been previously studied in different types of macrophages and experimental situations. Heinrich et al. focused on investigating the morphologic, phenotypic, and transcriptomic characteristics in canine blood-derived macrophages in vitro^31^. However, most studies have focused on murine^11,32,33^ or human origin^34,35^ both in pathological models and in models based on biomaterials and stiffness^8,11,13,35^. In all of them the relationship between changes in morphology and different phenotypes is demonstrated. In our study we showed that the activation or inactivation of the Ivns1abp gene is essential in this relationship during inflammation. Our study reflects that a pro-inflammatory environment inhibits the Ivns1abp gene in macrophages, so that primary functional roles such as phagocytosis or the ability to induce proliferation are compromised. Other features of Ivns1abp inhibition are changes in cell shape, reflecting modifications in the cytoskeleton, and polarization to the pro-inflammatory phenotype. In contrast, up-regulation of Ivns1abp induces polarization towards an alternative macrophage phenotype, significantly decreases the expression of pro-inflammatory mediators such as TNF-alpha and iNOS, and increases the expression of anti-inflammatory mediators such as IL-10 and mannose receptor. In addition, inflammatory polarization is prevented when encountering a pro-inflammatory environment. In other words, the Ivns1abp modulation would produce a change in the macrophage phenotype.

Since the cytoskeleton of macrophages is constantly changing both to adapt to different environments and to exert different innate functions, maintaining a stable cytoskeleton in a prolonged manner towards an anti-inflammatory and repair phenotype in a pro-inflammatory environment could give rise to the acceleration of repair and regeneration. In this regard, cell culture substrates designed to control cell shape have revealed that cell elongation and a stable cytoskeleton promote a healing phenotype, indicating that alterations in cell shape provide integral signals that modulate the polarization of macrophage phenotypes^11^. Furthermore, in recent years it has been shown that changes in cell shape influence the transcription of the phenotype genetic program in different cell types^36^ and also in macrophages^9^. Active phagocytosis is one of the main functions of alternately activated macrophages. These are dynamic processes that are mechanically linked to the rearrangement of actin filaments^37^. It is a complex process that continues to be studied and where the key challenge remains to understand how the signals affecting the cytoskeleton translate into a rearrangement of actin. In our study, the results in phagocytosis showed that an overregulation of Ivns1abp provides macrophages with a higher phagocytic capacity despite the presence of cytokines, suggesting that the mechanisms that impact on Ivns1abp in the macrophage will have a result in its phagocytic capacity. This is potentially interesting for pathological situations with deficient phagocytosis, such as in therapies against tumors, where the search for therapies continues to this day^38^.

By exploring the possible transcription factors that could modulate Ivns1abp in macrophages under inflammatory stimuli we focused on c-myc as the main candidate. C-myc is a proto-oncogene with unregulated expression that is associated with tumor development in mice and humans^39^. More recently, it has been shown to be involved in the induction of a large set of genes during the activation of alternative macrophages, either by direct interaction or indirectly through the induction of the signal transducer and transcription activator-6 (STAT6) and PPARγ^20^. C-myc is also regulated by cytokines^18,19,28^. In order to directly test the interaction of this transcription factor in Ivns1abp macrophages in a pro-inflammatory context, we evaluated whether cytokines or LPS modify the expression of c-myc/Ivns1abp. In addition, in the set of experiments we used the inhibitor c-myc 10058-F4 that specifically inhibits c-myc-Max interaction and prevents transactivation of the expression of the c-myc target gene. With this study, we demonstrated that inflammatory insults alter the activity of the c-myc transcription factor, modifying the expression of the Ivns1abp gene. We also confirmed the strong suppression of c-myc transcription by the pro-inflammatory stimulus described above by other authors^26,27^. Interestingly, inhibition of c-myc induced changes in cell morphology and a disruption of actin filaments forming clusters with tightly packed texture and a significant decrease in the levels of Ivns1abp expression. Such conclusions indicate the ability of c-myc to directly modulate the expression of Ivns1abp in these cells.

Lastly, given the properties of macrophages overexpressing Ivns1abp in actin cytoskeleton stability and resistance to inflammation and following our previous experience in cellular therapy with macrophages in renal pathology^5–7^, we hypothesized that these macrophages could be used as a treatment for damage associated with I/R injury. The experiments revealed that BMDMs that overexpress Ivns1abp were highly protective of renal function as well as the epithelial structure of renal tubules preventing mismatches of actin cytoskeleton loss. In addition, the repair observed after I/R injury and the decrease in inflammation leads us to postulate BMDMs overexpressing Ivns1abp as a potential tool to promote repair in the kidney versus I/R.

In conclusion, in this study we evidenced the central role of the lvns1abp gene in the promotion of alternative phenotypes and resistance to cytokines and the inflammatory environment, as well as its possible efficacy as a cell therapy approach. Our data reveals that pro-inflammation inhibits the Ivns1abp gene in macrophages, resulting in the loss of primary functions such as phagocytosis or the ability to proliferate. The consequences of Ivns1abp inhibition are also detected in cytoskeletal modifications and polarization to the pro-inflammatory phenotype. In contrast, preservation of Ivns1abp may induce an alternative activation of macrophages by providing resistance to inflammation.

## Supplementary information

Supplementary information.

## References

[1] Wang N, Liang H, Zen K (2014). Molecular mechanisms that influence the macrophage m1–m2 polarization balance. Front. Immunol..

[2] Das A (2015). Monocyte and macrophage plasticity in tissue repair and regeneration. Am. J. Pathol..

[3] Chinetti-Gbaguidi G, Staels B (2011). Macrophage polarization in metabolic disorders: functions and regulation. Curr. Opin. Lipidol..

[4] Liu YC, Zou XB, Chai YF, Yao YM (2014). Macrophage polarization in inflammatory diseases. Int. J. Biol. Sci..

[5] Jung M, Brüne B, Hotter G, Sola A (2016). Macrophage-derived Lipocalin-2 contributes to ischemic resistance mechanisms by protecting from renal injury. Sci. Rep..

[6] Guiteras R (2017). Macrophage overexpressing NGAL ameliorated kidney fibrosis in the UUO mice model. Cell. Physiol. Biochem..

[7] Guiteras R, Sola A, Flaquer M, Manonelles A, Hotter G, Cruzado JM (2019). Exploring macrophage cell therapy on Diabetic Kidney Disease. J. Cell. Mol. Med..

[8] Rostam HM (2016). The impact of surface chemistry modification on macrophage polarisation. Immunobiology.

[9] McWhorter FY, Davis CT, Liu WF (2015). Physical and mechanical regulation of macrophage phenotype and function. Cell. Mol. Life Sci..

[10] Dupont S (2011). Role of YAP/TAZ in mechanotransduction. Nature.

[11] McWhorter FY, Wang T, Nguyen P, Chung T, Liu WF (2013). Modulation of macrophage phenotype by cell shape. Proc. Natl. Acad. Sci. U. S. A..

[12] Gruber EJ, Leifer CA (2020). Molecular regulation of TLR signaling in health and disease: mechano-regulation of macrophages and TLR signaling. Innate Immun..

[13] Wosik J (2018). Magnetic field changes macrophage phenotype. Biophys. J..

[14] Sasagawa K (2002). Identification of Nd1, a novel murine kelch family protein, involved in stabilization of actin filaments. J. Biol. Chem..

[15] Inoue A (2005). Overexpression of Nd1-s, a variant form of new kelch family protein, perturbs the cell cycle progression of fibroblasts. DNA Cell Biol..

[16] Perconti G (2007). A The kelch protein NS1-BP interacts with alpha-enolase/MBP-1 and is involved in c-Myc gene transcriptional control. Biochem. Biophys. Acta.

[17] Saha A, Ahn S, Blando J, Su F, Kolonin MG, DiGiovanni J (2017). Proinflammatory CXCL12-CXCR4/CXCR7 signaling axis drives myc-induced prostate cancer in obese mice. Cancer Res..

[18] Liu T, Zhou S, Ko KS, Yang H (2015). Interactions between Myc and mediators of inflammation in chronic liver diseases. Mediators Inflamm..

[19] Liu H (2019). Inflammation-dependent overexpression of c-Myc enhances CRL4_DCAF4_ E3 ligase activity and promotes ubiquitination of ST7 in colitis-associated cancer. J. Pathol..

[20] Pello OM (2012). Role of c-myc in alternative activation of human macrophages and tumor-associated macrophage biology. Blood.

[21] Jung M (2018). Lipocalin-2 abrogates epithelial cell cycle arrest by PPARγ inhibition. Lab. Invest..

[22] Viñas JL, Hotter G, Pi F, Palacios L, Sola A (2007). Role of peroxynitrite on cytoskeleton alterations and apoptosis in renal ischemia-reperfusion. Am. J. Physiol. Renal Physiol..

[23] Mehta NN (2017). IFN-γ and TNF-α synergism may provide a link between psoriasis and inflammatory atherogenesis. Sci. Rep..

[24] Salim T, Sershen CL, May EE (2016). Investigating the role of TNF-α and IFN-γ activation on the dynamics of iNOS gene expression in LPS stimulated macrophages. PLoS ONE.

[25] Jin P (2016). Interferon-γ and Tumor Necrosis Factor-α Polarize bone marrow stromal cells uniformly to a Th1 Phenotype. Sci. Rep..

[26] Liu L (2016). Proinflammatory signal suppresses proliferation and shifts macrophage metabolism from Myc-dependent to HIF1α-dependent. Proc. Natl. Acad. Sci. U. S. A..

[27] Nakashima A, Kumakura S, Mishima S, Ishikura H, Kobayashi S (2005). IFN-alpha enhances TNF-alpha-induced apoptosis through down-regulation of c-Myc protein expression in HL-60 cells. J. Exp. Clin. Cancer Res..

[28] Dunham EE, Stevens EA, Glover E, Bradfield CA (2006). The aryl hydrocarbon receptor signaling pathway is modified through interactions with a Kelch protein. Mol. Pharmacol..

[29] Wolff T, O'Neill RE, Palese P (1998). NS1-Binding protein (NS1-BP): a novel human protein that interacts with the influenza A virus nonstructural NS1 protein is relocalized in the nuclei of infected cells. J. Virol..

[30] Zhang K (2018). Structural-functional interactions of NS1-BP protein with the splicing and mRNA export machineries for viral and host gene expression. Proc. Natl. Acad. Sci. U. S. A..

[31] Heinrich F (2017). Morphologic, phenotypic, and transcriptomic characterization of classically and alternatively activated canine blood-derived macrophages in vitro. PLoS ONE.

[32] Pelegrin P, Surprenant A (2009). Dynamics of macrophage polarization reveal new mechanism to inhibit IL-1beta release through pyrophosphates. EMBO J..

[33] Vereyken EJ (2011). Classically and alternatively activated bone marrow derived macrophages differ in cytoskeletal functions and migration towards specific CNS cell types. J. Neuroinflammation.

[34] Porcheray F (2005). Macrophage activation switching: an asset for the resolution of inflammation. Clin. Exp. Immunol..

[35] Lee HS (2013). Correlating macrophage morphology and cytokine production resulting from biomaterial contact. J. Biomed. Mater. Res. Part A.

[36] Lamouille S, Xu J, Derynck R (2014). Molecular mechanisms of epithelial-mesenchymal transition. Nat. Rev. Mol. Cell. Biol..

[37] Rougerie P, Miskolci V, Cox D (2013). Generation of membrane structures during phagocytosis and chemotaxis of macrophages: role and regulation of the actin cytoskeleton. Immunol. Rev..

[38] Feng M, Jiang W, Kim BYS, Zhang CC, Fu YX, Weissman IL (2019). Phagocytosis checkpoints as new targets for cancer immunotherapy. Nat. Rev. Cancer.

[39] Meyer N, Penn LZ (2008). Reflecting on 25 years with MYC. Nat. Rev. Cancer.

