# Supplementary material for: The influenza virus NS1A binding protein gene modulates macrophages response to cytokines and phagocytic potential in inflammation
Source: Sci Rep. 2020 Sep 17;10:15302. doi: 10.1038/s41598-020-72342-7 (PMC7498593; doi:10.1038/s41598-020-72342-7)
Supplement: Supplementary file 1 — Supplementary information. [file 41598_2020_72342_MOESM1_ESM.pdf]

SUPPLEMENTAL MATERIAL

The Influenza Virus NS1A Binding Protein gene modulates macrophages response to cytokines and phagocytic potential in inflammation

Georgina Hotter, Chrysoula Mastora, Michaela Jung, Bernhard Brüne, Teresa Carbonell, Claudia Josa, Juan Ignacio Pérez-Calvo, Josep Maria Cruzado, Roser Guiteras, Anna Sola*

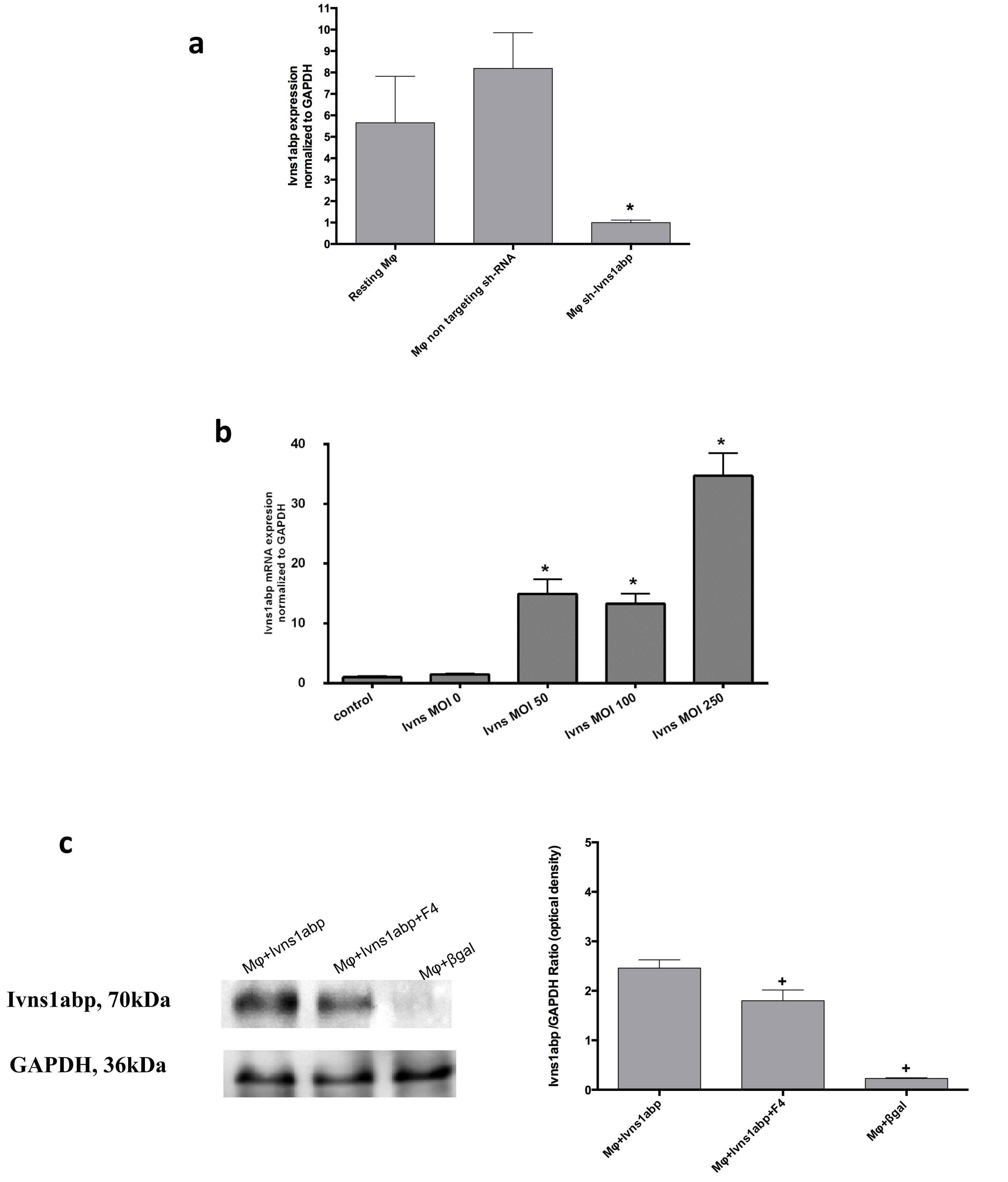

Supplementary data 1:

a) The efficiency in downregulation the Ivns1abp gene compared to the level of expression of untreated macrophages.
b) The efficiency of over-expression was measured by determining the mRNA by showing that the most effective concentration for the Ivns1abp vector is the MOI of 250. In this group expression of Ivns1abp gene was almost 35-fold up-regulated compared to the control β-gal group. Almost an 85% of the total cell number is transfected with this vector (data not shown).
c) Left, Representative image of Ivns1abp protein detection by Western Blot. Right, relative optical density of the protein detection

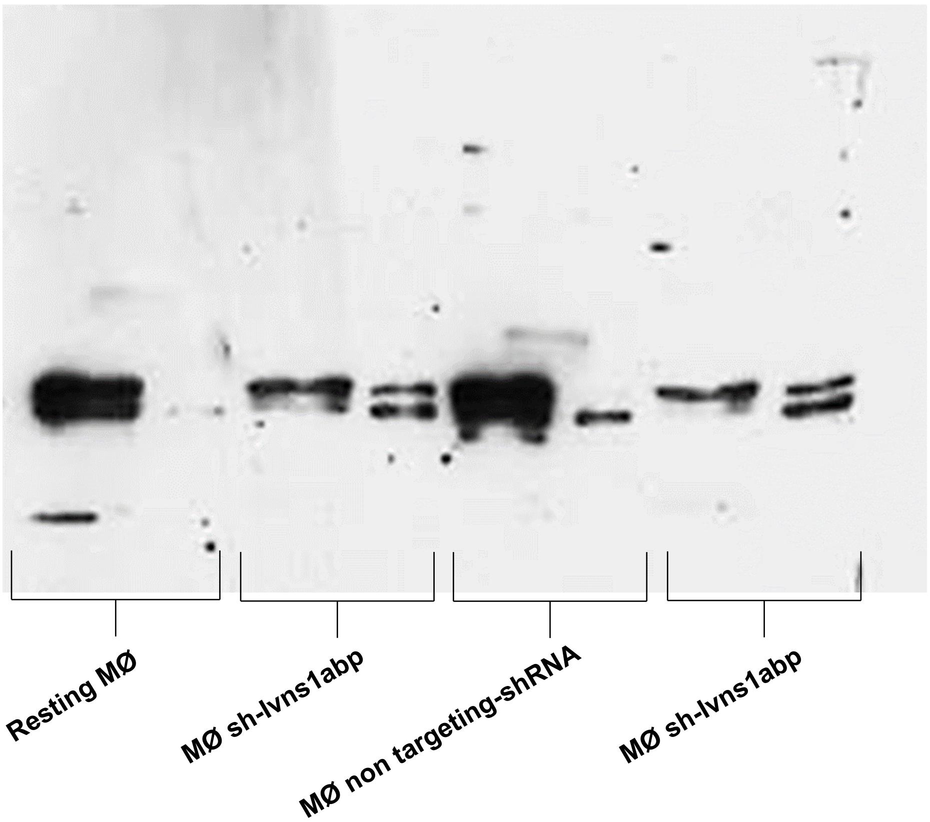

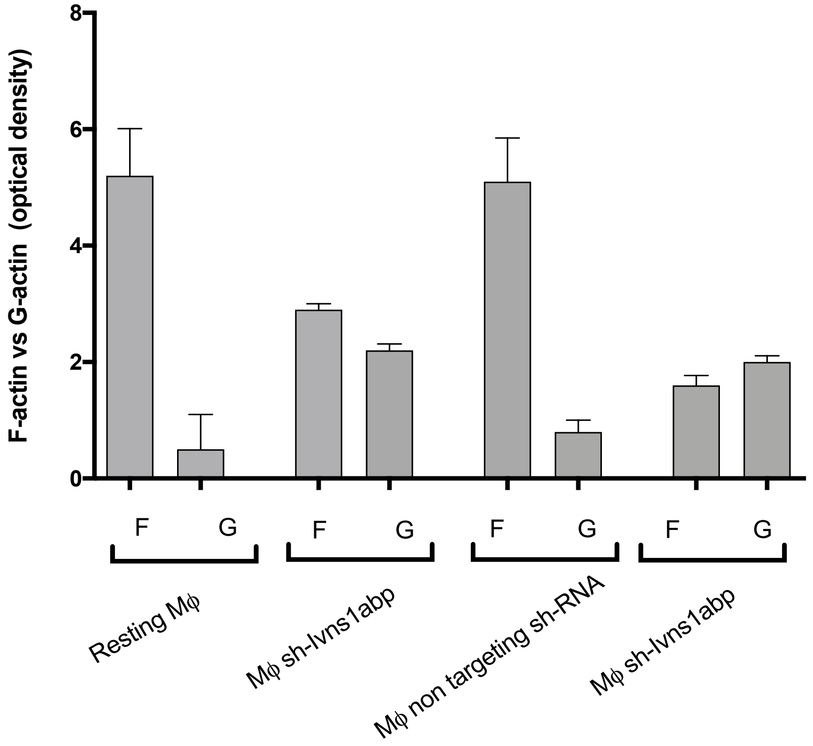

Supplementary information:

Full –length blot and densitometric analysis of protein bands corresponding to the cropped gel in figure 1
